# Transanal endoscopic microsurgery after the attempt of endoscopic removal of rectal polyps

**DOI:** 10.1007/s00464-022-09162-5

**Published:** 2022-03-04

**Authors:** Monica Ortenzi, Alberto Arezzo, Roberto Ghiselli, Marco Ettore Allaix, Mario Guerrieri, Mario Morino

**Affiliations:** 1 Department of General and Emergency Surgery, Polytechnic University of Marche, Via Conca 1, 60126 Ancona, Italy; 2 Department of Surgical Sciences, University of Torino, Turin, Italy

The incidence of rectal polyps has steadily increased in recent decades and will continue to rise. [1] The introduction of endoscopic screening programs has probably contributed to the improved detection of rectal polyps and early malignant lesions [2, 3]. With the aim to reduce morbidity and mortality of rectal surgery, in 1983, Gerhard Buess introduced Transanal Endoscopic Microsurgery (TEM) [4]. He conceived a novel endoscopic technology to facilitate the excision of rectal polyps through the anus [5]. This revolutionary technique enabled superficial or full-thickness excision of large adenomatous lesions. It soon became apparent that indications to TEM could be successfully extended to early malignant polyps [6, 7].

However, in the late nineties, endoscopy was advocated as a diagnostic technique and a therapeutic method. First, large piecemeal snare ablations were reported. Then, the use of endoscopic electrosurgical knives made it possible to achieve en bloc resection, known as Endoscopic Submucosal Dissection (ESD) [8–11]. The sharp increase in endoscopic resection of rectal polyps made the indications for TEM questioned [12].

This unresolved debate confuses the choice of the optimal treatment for complex rectal polyps. Concerns mainly arise where there is uncertainty around early malignancy or where complete resection of an adenomatous polyp is not obtained following endoscopic attempts [13–15]. Accurate prognostic information is not always available after endoscopic removal, mainly when the specimen is fragmented. [16] Additionally, fibrotic tissue growth at the polypectomy site could invalidate the already sub-optimal accuracy of pre-operative imaging techniques. Therefore, endoscopic ultrasound and/or Magnetic Resonance Imaging staging are often misleading [17]. The indication to resect the site of a previous endoscopic resection with a full-thickness technique has been recommended in cases of unexpected malignancy. However, the overall benefit remains unclear [13, 18].

This study aims to evaluate the outcomes of TEM following endoscopic resection of rectal polyps performed at two different centres, assess the value of further local excision, and identify features that may contribute to the decision-making process.

## Materials and methods

We merged two prospective databases and searched for all patients who underwent TEM following any endoscopic excision of a rectal polyp. The two databases include all consecutive patients who underwent TEM for any reason at the Department of Surgical Sciences of the University of Torino and the Department of Surgery of the University of Ancona. Altogether they consist of a total of 2520 patients performed between 1993 and June 2020. Databases consisted of Excel format sheets, including patient characteristics, pre-operative work-up, TEM indications, perioperative data, and follow-up until the latest contact. All the procedure were carried out by experienced surgeons with more than 100 procedures performed before the enrolment period.

A full-thickness excision was performed in all cases, and the rectal wall defect closed with an absorbable monofilament running suture secured with silver clips and, more recently, titan clips (Richard Wolf, Knittlingen, Germany). Two different sets were used: one was a dedicated instrumentation with a 12 or 20 cm modified rectoscope with 3 operative channels (Richard Wolf, Knittlingen, Germany) and 3D dedicated optics, the other a similar device 7 or 15 cm long (Karl Storz Endoskope, Tuttlingen) with standard 2D vision, both 4 cm in diameter. Both were fixed in position with a Martin arm, a three-elbowed device attached to the operating table. The traditional TEM curved instrumentation [19] or laparoscopic devices were used according to the preference of the surgeon.

We analysed the patients’ characteristics, pre-operative work-up, the type of surgery performed, the pre-operative data, the definitive histology, the intra-operative and post-operative complications and finally, the recurrence rate after treatment. The paper is written according to the STROCCS 2021 guidelines [20].

### Inclusion criteria

We enrolled all consecutive patients undergoing TEM after partial or complete endoscopic resection of a rectal polyp between January 2011 and June 2020. Indications for TEM were recorded as well as the number of previous endoscopic removal attempts. Common indications for TEM were the persistence of visible residual disease, both benign and malignant, and the histology of malignancy on the specimen even of an apparent complete endoscopic excision.

### Work-up

The need for radical rescue surgery was established based on post-TEM histology. All the patients fit for surgery and resulting affected by an early rectal cancer with unfavourable characteristics, or a locally advanced rectal cancer was considered eligible for radical abdominal surgery.

If the residual neoplasms or post-EMR scars were not easily detected at the rigid proctoscopy performed prior to TEM to correctly locate the lesions, patients underwent flexible sigmoidoscopy and tattooing before surgery.

TEM excision was considered curative in case of no residual malignant disease, excision of residual benign polyps or up to pT1sm1 cancers. In benign histology, these patients underwent post-operative follow-up every 3 months for 2 years, with clinical examination, digital rectal examination, flexible rectosigmoid endoscopy. In malignancy, follow-up was prolonged till 5 years after surgery, with 6 months intervals and included monitoring neoplastic markers and imaging for possible metastases. Patients with a follow-up shorter than 6 months were excluded. The analysis was conducted according to the intention-to-treat principle.

### Outcomes

The primary outcome was to assess the rate of curative TEM procedures avoiding the need for radical surgery. Secondary outcomes were morbidity at 30 days after TEM, rate of rescue surgery, recurrence rate, type of recurrence, overall survival and disease-free survival. Depending on the indication for TEM, results were analysed in three groups: (a) Complete endoscopic excision of unexpected cancers, (b) incomplete endoscopic excision of unexpected cancers, (c) incomplete endoscopic excision of adenomas.

### Statistical analysis

We summarised qualitative variables as absolute and percentage frequencies in brackets. We used Fisher’s exact test to evaluate differences between categorical variables. We reported the descriptive data as median with interquartile range (IQR) and Wilcoxon rank-sum test to compare them. Cox proportional-hazards regression was used to assess outcome and for univariate analysis. Univariate analysis was used to identify risk factors for incomplete endoscopic excision and recurrence. Results were expressed as odds ratios (OR) and 95% Confidence Intervals (95%CI).

A multivariate analysis Cox regression analysis was used to evaluate the prognostic role of clinical and surgical variables in recurrence probability. Hazard ratios (HR) and 95% Confidence Intervals (95%CI) were also calculated.

Gender, age (dichotomized at 60 years), distance from anal verge (dichotomized at 5 cm), tumour size before endoscopic removal (dichotomized at 2 cm), and tumour histological characteristics (benign, pT stage, differentiation, Lymph vascular invasion, budding) were considered as independent factors. The factors used in regression analyses were set a priori.

Kaplan–Meier estimates were obtained for local recurrence-free, disease-free and overall survival. Patients without recurrence were censored at the date of last follow-up or death.

The statistical significance was assessed at a level of probability of 0.05. All statistical analyses were performed using MedCalc Statistical Software (MedCalc Software bv, Ostend, Belgium).

## Results

We included a total of 150 patients who underwent TEM between January 2011 and June 2020. There were 103 males and 47 females (Table 1).

**Table 1:** Patients’ demographic and clinical characteristics

Variables	c-EMR	i-EMR	*p*	Total (*n* = 150)
	*n* = 77	*n* = 73		
Sex [Males, *n* (%)]	55 (71.4)	48 (65.7)	0.485*	103 (68.6)
Age [years, median (IRQ)]	68 (61–76)	69 (62–75)	0.749**	68.5 (62–76)
Size before endoscopy [cm, median IRQ)]	2 (1.5–3)	3 (2–4.2)	< 0.001**	3 (2–4)
Size > 2 cm [*n* (%)]	46 (59.7)	49 (67.1)	0.008*	95 (63.3)
Distance from anal verge [cm, median IRQ)]	7 (6–11)	9 (5–10.2)	0.709**	8 (6–11)
Site of the lesion [*n* (%)]				
Anterior	16 (20.8)	20(27.4)	0.444*	36 (32)
Right quadrant	16 (20.8)	11 (15)	0.401*	27 (18.7)
Left quadrant	19 (24.7)	20 (27.4)	0.714*	39 (26)
Posterior	26 (33.8)	18 (24.6)	0.726*	48 (32)
cT				
cT1	45 (58.4)	25 (34.2)	0.003*	70 (46.7)
cT2	8 (10.4)	0	0.006*	8 (5.3)
Surgical time [minutes, median (IRQ)]	45 (35–60)	45 (35–71.2)	0.224	45 (35–65)
Difficulty in dissection	3 (3.9)	4 (5.2)	0.713	7 (4.7)
Post-operative complications [*n* (%)]	7 (9.1)	11 (15.1)	0.318*	18 (12)
Dindo Clavien I [*n* (%)]	1 (1.3)	7 (9.6)	0.030*	8 (5.3)
Fever	1 (1.3)	4 (5.5)		
Bleeding	0	3 (4.1)		
Dindo Clavien II [*n* (%)]	4 (5.2)	2 (2.7)	0.681*	6 (4)
Bleeding	4 (5.2)	2 (2.7)		
Dindo Clavien IIIa [*n* (%)]	2 (2.6)	2 (2.7)	ns*	4 (2)
Suture dehiscence	1 (1.3)	1 (1.4)		
Bleeding	1 (1.3)	1 (1.4)	/	
Length of hospital stay [days, median (IRQ)]	3 (2.5–4)	3 (2–4)	0.945**	3 (2–4)
Readmissions [*n* (%)]	0	2 (3.4)	0.235*	2 ()
Rescue surgery [*n* (%)]	5 (6.5)	3 (4.1)	0.719*	8 (5.3)
Recurrences	4 (5.2)	4 (5.5)	ns*	(5.3)
Local	3 (3.9)	4 (5.5)	0.713*	7 (4.7)
Distant	1(1.3)	0	/	1 (0.7)

IRQ: 1st–3rd quartiles

*c-EMR* complete EMR, *i-EMR* incomplete EMR

*Fisher exact test

**Wilcoxon rank-sum test

Most rectal lesions were removed with a piecemeal endoscopic mucosal resection (p-EMR) (n = 133, 88.7%) and by ESD in the remaining 17 patients (11.3%). The median diameter of endoscopically excised lesions was 3 cm (IQR = 2–4 cm). The median distance of the lesions from the anal verge was 8 cm (IQR = 6–11 cm). Rectal lesions with size > 2 cm that underwent incomplete EMR were significantly larger than polyps that were excised entirely (median size 3 vs 2 cm, respectively, *p* = 0.001). All other considered variables were comparable (Table 1). The indication for TEM was primarily to obtain a wider local excision in cases of unexpected cancerous polyp observed on definitive histology following polypectomy (101, 67.3%) (Table 1).

In one case, the defect was left open due to the presence of an abscess following the endoscopic procedure. In 4 patients (2.6%), accidental opening of the peritoneum was observed during surgery. This was sutured transanally in all cases without sequelae. No intraoperative complications were reported. However, in 7 (4.7%) cases, an increased difficulty, defined as increased resistance to tissue dissection due to fibrosis, was reported. The median operative time was 45 min (IQR = 35–65 min,) and it did not statistically differ whether the lesion was excised entirely or not before TEM (*p* = 0.224) (Table 1).

Post-operative complications occurred in 18 patients (12%): 8 type I (5.3%), 6 type 2 (4%), 4 type 3a (2.7%) according to Dindo Clavien classification. The median length of hospital stay was 3 days (IQR = 2–4 days). Two patients (1.3%) were readmitted within 30 days of discharge, 1 for rectal bleeding and 1 for hyperthermia (Table 1).

### Results by pathology and staging

#### Complete endoscopic excision of unexpected cancers

**Fig. 1:**
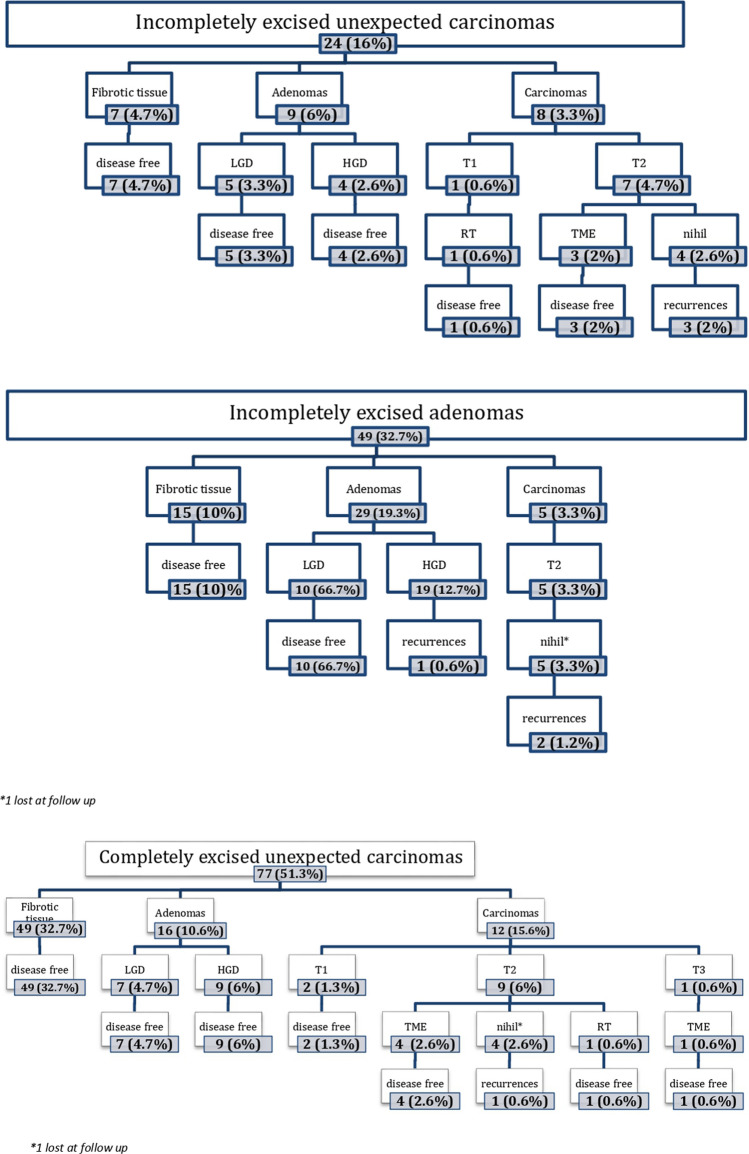
Results according to the completeness of endoscopic excision

Seventy-seven individuals received TEM after complete endoscopic excision of a polyp but unexpected harbour foci of malignant cells. A persistence of lesions in the form of dysplastic tissue was detected in 16 (20.8%) cases and of cancerous lesions in 12 (15.6%) (Fig. 1). In all cases, the lesions were defined as completely excised after TEM. No recurrences were observed after local excision of the residual dysplastic lesions. Pathology revealed pT1sm1 adenocarcinoma in 2 patients while showed a pT2 and pT3 infiltration of the rectal wall in 9 (10.4%) and 1 (8.3%) cases, respectively. Four T2 and the T3 adenocarcinomas underwent rescue Total Mesorectal Excision (TME), while the remaining refused or were deemed unfit for major surgery. The presence of fibrotic tissue was confirmed in 49 (63.6%) patients.

#### Incomplete endoscopic excision of unexpected cancers

**Fig. 2:**
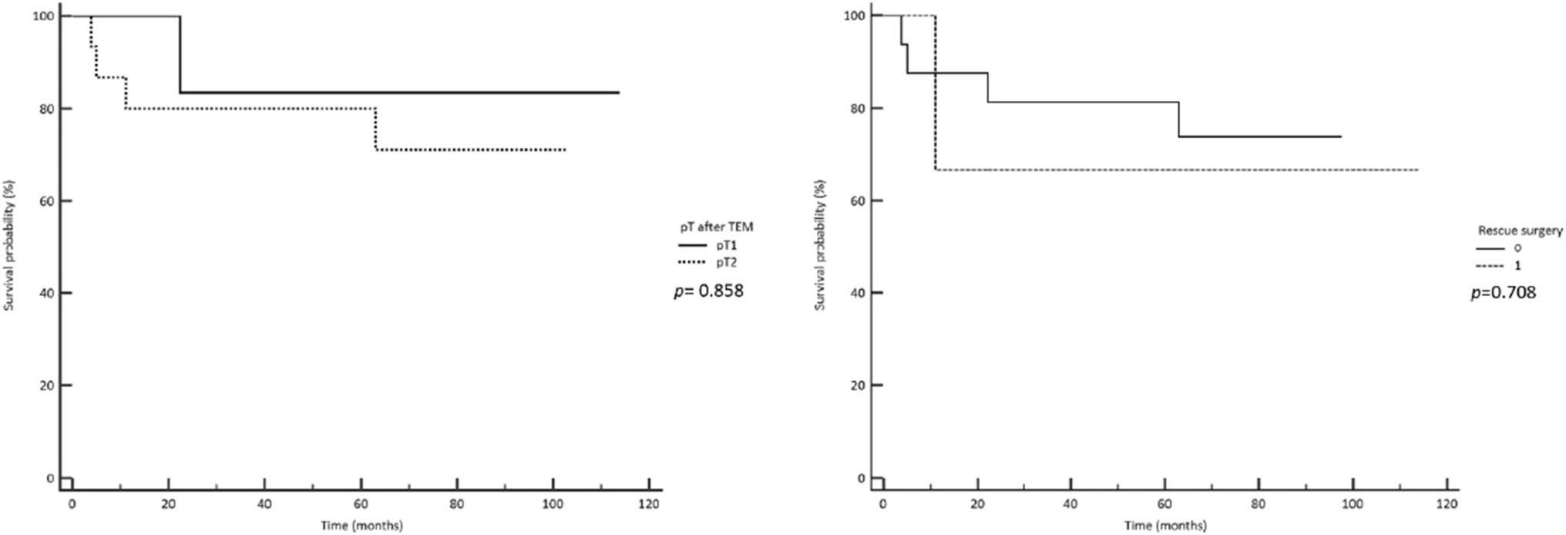
Disease-free survival probability according to pT stage after TEM and rescue surgery

Twenty-four individuals underwent TEM because of visible residual tissue after one or several attempts of endoscopic removal, with a histological report revealing the presence of unexpected cancerous cells (Table 2). The histological examination of the TEM specimen showed persistent cancer cells in 8 (33.3%) residual lesions, staged as pT1sm2 in 1 case and as pT2 in the remaining 7 cases, respectively (Fig. 2). The T1 adenocarcinoma was further treated with adjuvant radiotherapy after TEM and showed no recurrence at 23.4 months. Of the 7 patients diagnosed with pT2 cancer, 3 received a completion TME and were all free of disease at 9, 15 and 43 months, respectively. In contrast, 4 did not receive further treatment despite indications due to patients’ refusal or judged unfit for major surgery. Three of them experienced local recurrences at 6, 10 and 12 months, while the fourth is free of disease at 18 months. Nine patients (37.5%) had residual dysplastic tissue. None of these patients had a recurrence. In 7 (29.2%) patients, no residual disease was found.

**Table 2:** Results according to histology

Variables	Total (*n* = 150)	Post EMR malignant disease	Post EMR benign disease
		*n* = 101	*n* = 49
Gender [*n* (%)]			
Male	103 (68.7)	70 (69.3)	33 (67.3)
Female	47 (31.3)	31 (30.7)	16 (32.6)
Age [years, median (IQR)]	68.5 (62–76)	68 (61.5–76)	72 (61.7–76)
Distance from the anal verge [cm, median (IQR)]	7 (6–11)	7 (6–10)	
Macroscopic residual disease [*n* (%)]			
Yes	73 (48.7)	24 (23.8)	49 (100)
No	77 (51.3)	77 (76.2)	0
Post TEM histology			
Fibrosis	71 (47.3)	56 (55.4)	15 (30.6)
Adenomas [*n* (%)]	54 (36)	25 (24.7)	29 (59.2)
Low grade [*n* (%)]	22 (14.7)	12 (11.9)	10 (20.4)
High-grade dysplasia [*n* (%)]	32 (21.3)	13 (12.8)	19 (38.8)
Carcinomas (T) [*n* (%)]	25 (16.7)	20 (19.8)	5 (10.2)
T1 [*n* (%)]	4 (2.7)	4 (3.9)	0
T2 [*n* (%)]	20 (13.3)	15 (14.8)	5 (10.2)
T3 [*n* (%)]	1 (0.7)	1 (0.99)	0

IQR: 1st–3rd quartiles range

#### Incomplete endoscopic excisions of adenomas

Forty-nine individuals received TEM because of visible residual tissue after one or several attempts of endoscopic removal, with a histological report revealing the presence of adenomatous cells (Table 2). After local excision by TEM, definitive histology showed a pT2 residual adenocarcinoma in 5 (10.2%) individuals (Fig. 2). None of these underwent rescue TME, despite indication, due to patients’ refusal or because judged unfit for major surgery. In 29 (59.2%) patients, a residual adenomatous lesion was found. Of these, 1 of 19 patients with a high-grade dysplastic polyp recurred at 8.7 months after local excision, while there was no recurrence in the 10 patients with a low-grade dysplastic adenoma. Only fibrotic tissue was found in the remaining 15 (30.6%) patients.

#### Follow-up of local excision by TEM of unexpected cancer

Overall, 25 patients were diagnosed with residual or recurrent cancer on the TEM specimen. Three were diagnosed with pT1 cancer, 21 with a pT2 and 1 with a pT3, respectively. With a minimum follow-up of 6 months, the disease-free survival (DFS) was 89.9% (95% CI 78.4–99.4, *p* = 0.319). Rescue radical surgery was performed in 8 patients (5.3%) (Table 1) and consisted of TME in 6 cases, TransAnal TME (TaTME) in 1 case and Abdominal Perineal Resection (APR) in 1 case. Definitive histology showed remnant disease in 5 cases, staged pT3N0 in 3, pT2 N2a in 1, and pT0 N1 in 1 case, respectively. None of them demonstrated recurrent disease at a minimum follow-up of 7 months (median 78 months, IQR = 54–101 months). No recurrence was observed among patients staged as pT1 cancer. Thirteen of the 21 patients diagnosed with pT2 cancer after TEM did not undergo rescue surgery, either due to refusal or judged unfit. While no recurrence was observed among those who underwent further radical surgery, an overall recurrence rate of 46.1% was reported in the rest of the patients (6/13). The relapse of neoplastic tissue occurred in 1 case as dysplasia, which was further treated with TEM, and in 6 patients as carcinoma treated in 2 cases with TME, in 1 case with APR, in 1 further case TEM and 1 case with palliative radiotherapy (Table 3) while a seventh patient refused further treatment. One patient, after rescue surgery by APR, developed pulmonary metastases. The univariate analysis for risk of recurrence (Table 4) showed no statistical significance for gender and distance from the anal verge. Size before endoscopy and malignant histology before TEM were demonstrated to be significant risk factors for recurrence. The multivariate analysis for risk of recurrence (Table 5) indicated size before endoscopy as an independent predictor of recurrence (Fig. 2).

**Table 3:** Pattern of recurrence

Patients	Post EMR histology	Indication to TEM	Post TEM histology	Time to recurrence	Management of recurrence	Follow-up
1	Adenocarcinoma	Radicalization of complete excision of unexpected malignancy	pT2	8.4 months	TME (pT4b N0)	Pulmonary metastasis at 34.8 months
2	HGD	Radicalization of three previous attempts of removal	pT2	5 months	TEM (HGD)	Free of disease
3	HGD	Radicalization of incomplete excision	HGD	8.7 months	TEM (HGD)	Free of disease
4	Adenocarcinoma	Radicalization of complete excision of unexpected malignancy	pT2	23 months	RT	Free of disease
5	Adenocarcinoma	Radicalization of incomplete excision	pT2	3.74 months	/	Lost after local recurrence
6	Adenocarcinoma	Radicalization of complete excision of unexpected malignancies	pT2	10.4 months	TME (pT2 N0)	Free of disease
7	Adenocarcinoma	Radicalization of incomplete excision of unexpected malignancy	pT2	3.8 months	APR (pT3 N0)	Second recurrence at 36 months treated with RT

*HGD* high grade dysplasia, *TME* total mesorectal excision, *APR* abdominal perineal resection, *CRT* chemoradiotherapy, *RT* radiotherapy

**Table 4:** Univariate analysis of categorical variables as risk factors of recurrence

Variables	OR	95% CI	*p*
Gender [Male vs female]	0.340	0.039–2.909	0.324
Age [< 60 years vs ≥ 60 years]	0.514	0.093–2.821	0.464
Distance from anal verge [< 5 cm vs ≥ 5 cm]	2.144	0.249–18.489	0.450
Size before endoscopy [< 2 cm vs ≥ 2 cm]	6.160	1.248–30.406	0.037
Endoscopic excision [Complete vs incomplete]	1.533	0.329–7.126	0.583
Histology [Adenoma vs carcinoma]	17.656	0.157–98.739	< 0.001
pT stage [pT1 vs pT2]	0.615	0.043–8.703	0.724
Tumour differentiation [G1 vs G2]	/	/	0.055
Lymph vascular invasion [LV1 vs LV0]	/	/	0.302
Tumour budding [Budding+ vs budding−]	/	/	0.094

**Table 5:** Factors associated with recurrence on multivariate analysis

	95%CI			
	OR	Lower	Upper	p*
Gender [M vs F]	0.46	0.05	3.91	0.445
Age > 60 vs age ≤ 60 years	0.53	0.10	2.71	0.471
Distance from anus (cm)	0.49	0.25	17.2	0.457
Size before endoscopy (cm)	1.41	0.27	7.20	0.674
T1 vs T2	0.61	0.05	6.73	0.702

*Cox regression model

## Discussion

When introduced in 1983, TEM offered both a minimally invasive technique and a definitive treatment for large rectal polyps. This is because it enabled an en-bloc polyp resection through either a full-thickness or a submucosal rectal wall excision [9–11]. More recently, p-EMR and ESD became increasingly adopted to manage rectal polyps. Consequently, the use of TEM has been questioned and, in some cases, even deemed obsolete. Nevertheless, the optimal management of these polyps remains highly debated for several reasons [18, 21–25]. Not rarely TEM is advocated after incomplete endoscopic resection or the discovery of unexpected cancer.

As always, TEM, even after a previous endoscopic attempt, aims at reducing the need for major surgery (TME or APR), which is burdened with a high rate of complication and impaired quality of life. Some of the observed results were predictable. The short operative time and the low morbidity align with the results obtained when TEM is used as first-line treatment. Similar to standard TEM results are intraoperative and post-operative complications, all minor and manageable with no further intervention.

Other findings were less obvious and deserved discussion. About 1/3 of TEM procedures performed after apparently complete removal of unexpected cancer and 2/3 of those performed after incomplete removal of an unexpected cancer show persistent neoplastic tissue. This is malignant in about 1/6 in the first case and about 1/3 in the second case. Still, TEM showed excellent characteristics to select those patients who deserve rescue surgery, saving the vast majority of patients (81/101, 80%) with no indication based on the pathology examination of the specimen resected by TEM. Moreover, even after incomplete endoscopic excision of a histology proven adenoma, there is a 10% risk to find malignant tissue at further local excision. Fortunately, in all these cases, TEM has been deemed an appropriate intermediate treatment with this indication.

Correct initial staging is essential to provide a precise indication for radical surgery [15, 26]. This is very difficult to obtain at first observation of a rectal polyp, with about 20% overstaging and about 20% understaging of endoscopic ultrasound (EUS) on T (personal findings). Magnetic Resonance Imaging (MRI) performs even worse when studying early rectal cancers. In the study of early rectal cancers, MRI has the unique role in determining the possible N+ status, with a slight advantage in the sensitivity compared to EUS, nevertheless not overcoming 50% in many series [27–29]. Things get even worse once an endoscopic removal has been attempted. The possibility to identify the different wall layers correctly becomes hazardous, reducing further reliability. In this scenario, TEM gains importance as a diagnostic tool, even earlier than a possible treatment.

Based on the histology of the TEM specimen, we were able to reserve radical surgery only for neoplasm at high-risk of recurrence for a remnant disease of invasive carcinoma. At the same time, TEM was curative in early malignancies since none of the T1 tumours recurred. Some could argue that the performance of TEM after a complete endoscopic excision could jeopardise oncological results, being even redundant if high-risk features are detected in the endoscopic samples and delaying the execution of radical surgery. [15] In the present study, 25/101 (16.7%) patients with a previous cancer diagnosis needed a rescue TME, in line with the literature [6]. Only 8 underwent radical surgery and 2 RT. In 2 cases, rescue surgery found metastatic lymph nodes though no residual malignant cells were found in the rectal wall. However, our results demonstrate that rescue surgery can be effectively performed after TEM excision. Similarly, repeated unsuccessful attempts at the endoscopic resection of rectal polyps could delay appropriate staging and raise the risk of overlooking a residual malignancy.

Our data highlighted an interesting recurrence pattern since no statistical difference in the recurrence rate between complete and incomplete EMR was observed. Notably, we found that 10.2%, 5/49 with a previous adenoma diagnosis and macroscopic residual disease, had a locally advanced rectal cancer, and 40% requiring further surgery. It is possible that the excision of the previous polypectomy scar could have prevented further recurrence.

All the T1 tumours in our series were free of recurrence, avoiding major abdominal surgery, although it would have been mandatory in the unexpected case. As expected, a 40% recurrence rate was observed in T2 tumours that did not undergo radical surgery, suggesting that TEM alone could not be considered curative in these patients but has a diagnostic role alone. Radical surgery (TME) was always possible following TEM with no particular difficulty reported by the operators, providing a curative treatment when indicated.

Finally, our data support previously published data in questioning the use of endoscopic resection for rectal lesions over 2 cm [12, 15, 30, 31]. Indeed, in several cases when the initial endoscopic excision was deemed complete, TEM was subsequently indicated due to unexpected findings of either carcinoma or persistent dysplastic tissue in the TEM specimen on histological examination, with dysplastic cells seen in 21% and malignant cells in 16%.

Based on these findings, endoscopic resection may be an inappropriate treatment modality for large rectal polyps, not only for staging but also as an excisional technique due to the possibility of unexpected malignancy. As expected, a statistical significance in lesion size before endoscopic removal was observed between complete and incomplete EMRs, suggesting that larger size could be an independent factor support TEM as the primary treatment modality for such rectal polyps. [27] No risk factor for locally advanced rectal cancer recurrence was statistically significant in our series, probably due to the small number of cases. However, size before EMR demonstrated a risk factor for recurrence in both univariate and multivariate analyses. Therefore, in our opinion, a rate of almost 30% residual malignant cells justifies performing a completion TEM, despite the 50% rate of negative histological findings.

In conclusion, TEM represents an appropriate alternative treatment modality to repeated endoscopic excision and offers therapeutic completion in incomplete treatment with endoscopic therapy. Although minimally invasive, this study confirms that TEM avoids radical surgery in the majority of patients with suspected residual disease following endoscopic excision, which in turn facilitates organ preservation and improves patient quality of life in the majority of cases.
